# Influenza A Virus Inhibits Type I IFN Signaling via NF-κB-Dependent Induction of SOCS-3 Expression

**DOI:** 10.1371/journal.ppat.1000196

**Published:** 2008-11-07

**Authors:** Eva-K. Pauli, Mirco Schmolke, Thorsten Wolff, Dorothee Viemann, Johannes Roth, Johannes G. Bode, Stephan Ludwig

**Affiliations:** 1 Institute of Molecular Virology (IMV), Centre of Molecular Biology of Inflammation (ZMBE), WWU Muenster, Germany; 2 Robert-Koch-Institute (RKI), Berlin, Germany; 3 Institute of Immunology and Department of Pediatrics, WWU Muenster, Germany; 4 Interdisciplinary Center for Clinical Research (IZKF), UKM Muenster, Germany; 5 Clinic for Gastroenterology, Hepatology und Infectiology, HHU Düsseldorf, Germany; Mount Sinai School of Medicine, United States of America

## Abstract

The type I interferon (IFN) system is a first line of defense against viral infections. Viruses have developed various mechanisms to counteract this response. So far, the interferon antagonistic activity of influenza A viruses was mainly observed on the level of IFNβ gene induction via action of the viral non-structural protein 1 (NS1). Here we present data indicating that influenza A viruses not only suppress IFNβ gene induction but also inhibit type I IFN signaling through a mechanism involving induction of the suppressor of cytokine signaling-3 (SOCS-3) protein. Our study was based on the observation that in cells that were infected with influenza A virus and subsequently stimulated with IFNα/β, phosphorylation of the signal transducer and activator of transcription protein 1 (STAT1) was strongly reduced. This impaired STAT1 activation was not due to the action of viral proteins but rather appeared to be induced by accumulation of viral 5′ triphosphate RNA in the cell. SOCS proteins are potent endogenous inhibitors of Janus kinase (JAK)/STAT signaling. Closer examination revealed that SOCS-3 but not SOCS-1 mRNA levels increase in an RNA- and nuclear factor kappa B (NF-κB)-dependent but type I IFN-independent manner early in the viral replication cycle. This direct viral induction of SOCS-3 mRNA and protein expression appears to be relevant for suppression of the antiviral response since in SOCS-3 deficient cells a sustained phosphorylation of STAT1 correlated with elevated expression of type I IFN-dependent genes. As a consequence, progeny virus titers were reduced in SOCS-3 deficient cells or in cells were SOCS-3 expression was knocked-down by siRNA. These data provide the first evidence that influenza A viruses suppress type I IFN signaling on the level of JAK/STAT activation. The inhibitory effect is at least in part due to the induction of SOCS-3 gene expression, which results in an impaired antiviral response.

## Author Summary

The type I interferon (IFN) system is one of the most powerful innate defenses against viral pathogens. Most RNA viruses are sensitive to the action of type I IFN. Therefore, these pathogens have evolved strategies to evade this response. For example, influenza viruses express a viral protein, the non-structural protein 1 (NS1), that suppresses production of IFNβ by lowering cellular sensitivity to viral nucleic acid as a pathogen pattern. Here we present data indicating that influenza A viruses are not only capable of suppressing production of the IFNβ gene but also inhibit action of this antiviral cytokine on cells. This occurs by viral induction of a cellular protein, the suppressor of cytokine signaling (SOCS)-3, a potent endogenous inhibitor of IFN signaling. This is a novel mechanism by which influenza viruses inhibit the antiviral response of the host and paves the path to efficient virus replication. This may be especially relevant for influenza viruses that induce high cytokine responses (cytokine burst), such as highly pathogenic avian influenza viruses of the H5N1 subtype. Induction of SOCS-3 expression would allow efficient replication despite high IFN and cytokine levels.

## Introduction

Influenza A viruses are negative-stranded RNA viruses that belong to the family of orthomyxoviruses. The segmented genome of influenza A virus encodes for up to 11 viral proteins. As many other viruses, influenza viruses have evolved strategies to counteract cellular antiviral responses, especially to circumvent the type I IFN system as a first line of defense against the pathogenic invader.

Among the influenza viral proteins, the NS1 has been identified as the main type I IFN antagonistic factor. So far two major mechanisms have been described by which NS1 suppresses the initial expression of IFNβ. On the one hand NS1 inhibits vRNA-mediated induction of the transcription factors interferon regulatory factor-3 (IRF-3), activating protein–1 (AP-1) and NF-κB that target the IFNβ promoter. This most likely occurs via binding to the RNA-sensor retinoic acid inducible gene (RIG-I) and inhibition of RIG-I-mediated signaling in response to viral RNA [1],[2]. On the other hand NS1 inhibits maturation [3],[4] and nuclear export of host mRNAs [5]. Other functions of the multifunctional protein include block of activation of the dsRNA-activated protein kinase PKR by direct interaction [6] or activation of the phosphatidylinositol-3 kinase PI3K/Akt pathway to prevent premature apoptosis induction [7],[8].

While the NS1-mediated antagonistic activities of influenza viruses mainly affect the induction of genes such as IFNβ, so far no viral suppression of IFN signaling has been described.

IFN are among the first molecules synthesized in response to viral infections [9]. The IFN family includes three classes. Type I comprises the well known IFNα and IFNβ. The only member of type II IFN is IFNγ. Type III IFN comprises IFNλ1, -λ2, and -λ3. All classes of IFN bind to different receptors and are structurally not related [10],[11]. Type I IFN belong to the key cytokines produced by influenza A virus-infected epithelial cells [12],[13]. The antiviral activity of type I IFN is mediated by a set of IFN-induced genes (ISGs).

Binding of IFNα/β to its receptor is the initial step in this signaling process, followed by activation of the JAK family and subsequent activation of STAT proteins [14].

Ligand binding leads to dimerisation of the type I IFN receptor subunits IFNAR1 and IFNAR2 and causes their conformational change. The JAK kinase Tyk2, which is constitutively bound to IFNAR1, phosphorylates the receptor at tyrosine residues and creates a docking site for STAT2. Subsequently, Tyk2 phosphorylates STAT2 at Y690. At the same time the receptor-bound JAK1 phosphorylates STAT1 at Y701 [15],[16].

The phosphorylated transcription factors dimerise and bind to IRF-9 [17]. The newly formed heterotrimer, called IFN-stimulated gene factor 3 (ISGF3), translocates into the nucleus and binds to IFN-stimulated response elements (ISRE), to initiate gene transcription of ISGs. Treatment of cells with type I IFN up-regulates expression of an array of genes including SP110, IRF-1 and many others [18]. Among these ISGs the 2′, 5′ -oligoadenylate synthetase 1 (OAS1), the Mx proteins and the dsRNA-activated protein kinase (PKR) are described to directly interfere with viral replication [19]. Both, PKR and the OAS1/RNaseL system are capable of inhibiting cellular and viral translation.

IFN-induced JAK/STAT signaling can be inhibited at different levels by several viral and cellular factors through various mechanisms. The large T-antigen of murine polyomavirus (MPyV) binds to JAK1 and inhibits downstream signaling [20], whereas the VP24 of Ebola virus (EBOV) binds to karyopherina-1 thereby blocking nuclear accumulation of STAT1 [21].

Endogenous cellular key regulators, capable of negatively regulating JAK/STAT-mediated signal transduction, include suppressor of cytokine signaling (SOCS) proteins, protein tyrosine phosphatases (PTP) and protein inhibitor of activated STATs (PIAS). The family of SOCS proteins comprises eight members (cytokine-inducible SH2 domain-containing protein (CIS) and SOCS1-7). All members contain a central SH2 domain, an N-terminus of variable length and sequence and a C-terminal 40 amino-acid module called SOCS box [22]. The SOCS box is necessary for recruitment of the ubiquitin transferase system and for stabilization and/or degradation of SOCS proteins [23]–[25]. The N-terminus contains a kinase inhibitory region (KIR), which functions as pseudo substrate for the JAK [26]. SOCS-1 and SOCS-3 differ in their mode of action. For inhibition of the kinase activity of JAKs, SOCS-1 binds directly to the activation loop of JAKs [26]–[28]. In contrast, SOCS-3 first binds to the receptor [29],[30].

Induction of SOCS-3 gene transcription by viruses was reported for HSV-1, HCV [31]–[33] and for respiratory viruses, such as SARS and RSV [34],[35]. The level of induction of SOCS-3 by HSV-1 seems to determine whether infection turns to acute or persistent progression [31]. For HCV it has been suggested that upregulation of SOCS-3 may contribute to the non-responsiveness of HCV patients to IFN therapy [33], [36]–[38]. Elevated SOCS-3 mRNA levels during RSV infection were linked to Th2 cell-mediated immune disease as atopic dermatitis and asthma [39],[40].

In the present study we show that influenza A virus can be added to the list of viruses that induce SOCS-3 expression. The protein functionally interferes with viral replication by providing a virus-supportive IFN-antagonistic activity on the level of type I IFN-signaling that has not been described so far.

## Results

### IFNα/β but not IFNγ-induced STAT phosphorylation is inhibited in influenza A virus infected cells

**Figure 1:**
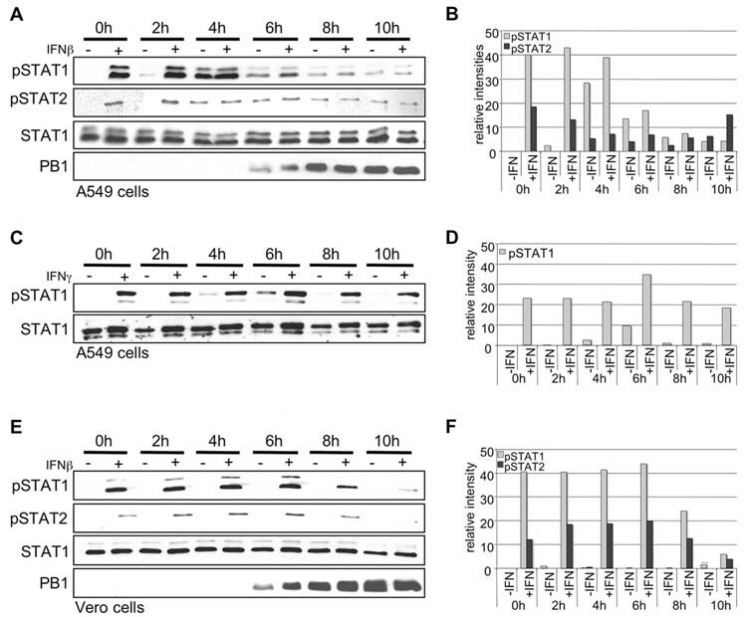
Influenza virus infection results in impaired IFNβ-induced STAT1 and STAT2 phosphorylation. A549 (A, C) or Vero cells (E) were infected with the human influenza virus PR8 (H1N1) (MOI = 5) for the indicated time points and were subsequently stimulated for 15 min with either human IFNβ at a concentration of 100 U/ml (A) or 500 U/ml (E) or human IFNγ 500 U/ml (C). Cells were lysed and cell extracts were separated by SDS-PAGE and blotted onto nitrocellulose membranes. Membranes were incubated with anti-phospho-STAT1, anti-STAT1, anti-phospho-STAT2 and anti-PB1 antibodies in Western blots. (B, D, F) Quantification of relative pSTAT1 and pSTAT2 band intensities in A, C and E using AIDA software and 2D densitometry (Fuji).

**Figure 2:**
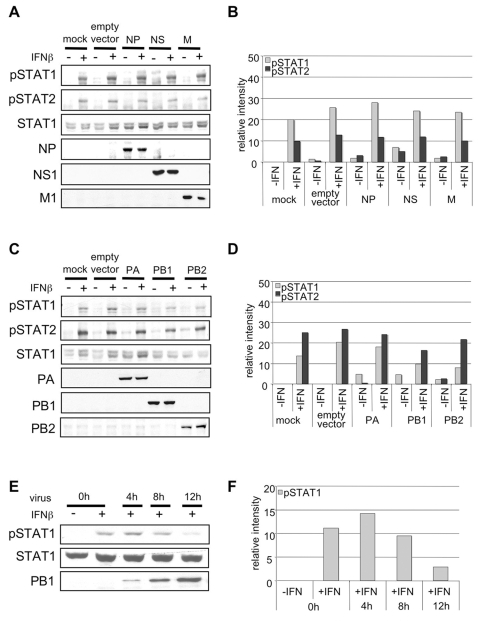
Forced expression of influenza viral proteins does not result in impaired IFNβ-induced STAT1 and STAT2 phosphorylation. HEK293 cells were transfected with 500 ng plasmid DNA for expression of viral NP, M, NS, (A) PA, PB1 and PB2 (C) genes (see Table 1 for accession numbers of viral genes) using L2000 according to manufacturer's instructions. Note that the Pol II constructs in use also give rise to expression of second reading frames in the NS, M and PB1 genes (NS2, M2, PB1-F2). 48 h post transfection cells were stimulated with human IFNβ (500 U/ml) for 15 minutes. Total protein lysates were subjected to Western blot analysis using anti-phospho-STAT1, anti-phospho-STAT2, anti-STAT1 antibodies. Expression of influenza viral proteins was monitored with antibodies against NP, M1, NS1, PA, PB1 or PB2. (E) HEK293 cells were infected with the human influenza virus PR8 (H1N1) (MOI = 5) for the indicated time points and were subsequently stimulated for 15 min with either human IFNβ at a concentration of 100 U/ml. Cell lysates were subjected to Western blots as described. (B, D, F) Quantification of relative pSTAT1 and pSTAT2 band intensities in A, C and E using AIDA software and 2D densitometry (Fuji).

Phosphorylation of STAT1 and STAT2 by members of the JAK tyrosine kinase family is a prerequisite for activation of these transcription factors to drive type I IFN-induced gene expression. Therefore, we analyzed whether STAT phosphorylation patterns are altered in influenza A virus infected cells that were stimulated with IFN at different time points post infection (p.i.). The human alveolar epithelial cell line A549 was infected with the influenza A virus strain A/Puerto-Rico/8/34 (H1N1) (PR8) (Figure 1A). Cells were subsequently stimulated with IFNβ at given time-points p.i. and STAT phosphorylation was assessed in Western blots. Both STAT1 and STAT2 were readily phosphorylated upon cytokine stimulation in uninfected cells or in infected cells up to 4 h p.i. (Figure 1A). Furthermore, virus infection alone resulted in a significant induction of STAT phosphorylation 4–6 h p.i., presumably caused by virus-induced IFN expression. However, at later time points (6–10 h p.i.), in A549 cells both virus- and IFN-induced STAT1 and STAT2 phosphorylation was markedly reduced (Figure 1A). Similar patterns were observed upon stimulation of cells with IFNα or upon infection with other viruses, such as the human influenza virus A/Victora/3/75 (H3N2) (data not shown). In addition, this phenomenon could also be detected in other epithelial cells such as the human embryonic kidney cell line HEK293 (Figure 2E) or the human umbilical vein endothelial cells (HUVEC) (Figure S1B). Inhibition was not caused by indirect disturbing effects on cellular metabolism or enzyme activities due to ongoing virus replication, since IFNγ-induced STAT1 phosphorylation was not affected at all (Figure 1C). Finally, involvement of any auto- or paracrine action of virus-induced type I IFN could be ruled out, as the inhibitory effect was also observed in Vero cells lacking functional type I IFN genes (Figure 1E).

### Forced expression of influenza virus proteins does not result in reduced STAT1 phosphorylation

With regard to the molecular basis of impaired IFNα/β-induced STAT phosphorylation in infected cells it was striking that the inhibitory effect correlated with the accumulation of viral proteins, as monitored in PB1 Western blots (Figures 1A and 1E). Thus, the question arose whether individual expression of viral proteins may result in the interference with STAT1 phosphorylation. Out of the 11 viral proteins of PR8 we choose the nucleoprotein (NP), the NS1 protein, the matrix protein (M1) (Figure 2A) and the subunits of the viral polymerase, PA, PB1 and PB2 (Figure 2C), for a representative experiment. These proteins are known to bind to vRNA/RNPs or to interfere with the RNA-mediated innate immune response. For efficient transfection of the expression constructs we used the highly susceptible cell line HEK293 that also exhibits impaired IFNβ-induced STAT1 phosphorylation at later stages of infection (Figure 2E). 24 h post transfection cells were stimulated with IFNβ and STAT phosphorylation was monitored in Western blots (Figures 2A and C). Expression of none of the viral proteins resulted in a significant decrease of IFNβ-induced STAT1 or STAT2 phosphorylation (Figures 2A and 2C). Similar results were obtained in the human bronchial epithelial cell line H1299 when expressing M1, NS1 or NP alone or in different combinations (data not shown). Thus, we concluded that viral proteins most likely do not play a prominent role as blockers of IFNα/β-induced JAK/STAT signaling.

### Impaired STAT1 phosphorylation is not mediated by virus-induced phosphatases

**Figure 3:**
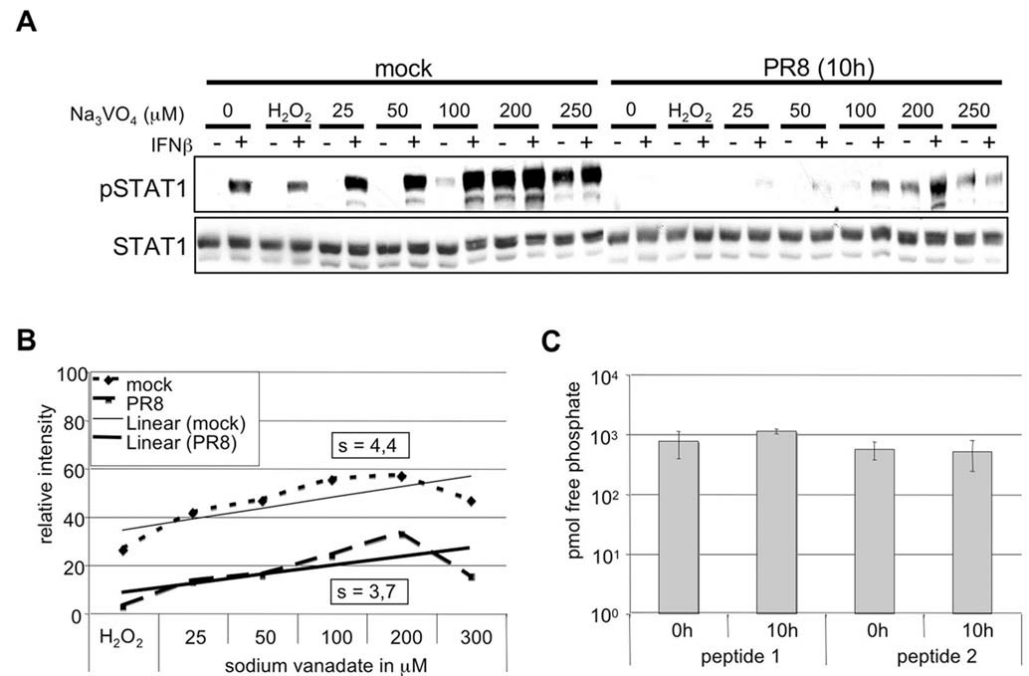
Phosphatases do not mediate inhibition of IFNβ-induced STAT1 phosphorylation in infected cells. (A) Vero cells were infected for 10 h with PR8 (MOI = 5) or left uninfected. Prior stimulation with human IFNβ (500 U/ml for 15 min), cells were treated for 10 min with sodium vanadate at concentrations indicated. Cells were harvested and protein lysates were subjected to Western blot analysis using anti-phospho-STAT1 and anti-STAT1 antibody. H_2_O_2_: was used as a control for solvent conditions. (B) Quantification of band intensities in (A). To visualize the effect of sodium vanadate on the STAT1 phosphorylation in infected and uninfected cells, band intensities of IFNβ stimulated samples were determined relative to background. Linear regression was calculated using the Excel software (Microsoft) (s = slope of the regression line). (C) Phosphatase activity in A549 cells infected wit PR8 (MOI = 5) was determined using tyrosine phosphatase assay (Promega) according to manufacturers instructions. For measurement of newly generated free phosphate two different phosphorylated pseudosubstrates (peptide 1 and peptide 2) were used.

Decrease of STAT phosphorylation might also be due to the action of virus-induced phosphatases. On the one hand these enzymes may cause direct dephosphorylation of STAT proteins. On the other hand phosphatases could act via an indirect mechanism by dephosphorylation and inactivation of JAKs resulting in an attenuated phosphorylation of STATs. Several protein tyrosine phosphatases (PTPs) are known to mediate dephosphorylation of both, JAKs and STATs [41]. In order to investigate whether influenza A virus activates phosphatases that subsequently target JAKs or STATs, we treated infected or uninfected A549 cells with the well-known tyrosine phosphatase inhibitor sodium vanadate [42],[43]. Uninfected cells or cells infected with PR8 for 10 h were incubated with increasing amounts of this compound 10 min prior to stimulation with IFNβ. This time point of infection was chosen since we observed considerable inhibition of IFN-induced STAT1 phosphorylation in the course of infection (Figures 1A and 1E). Increasing concentrations of vanadate lead to a gradual shift of the steady state balance of phosphorylation/dephosphorylation. Accordingly, a gradual increase of STAT1 phosphorylation was observed that was similar in both infected and uninfected cells, albeit starting from different basal levels of phospho-STAT1 (Figures 3A and B). This is illustrated by an almost identical slope of the regression line in the graphical analysis of the band intensities of the IFNβ stimulated samples (Figure 3B). If the blockade of IFNβ-induced STAT1 phosphorylation would be mediated by specific virus-activated phosphatases, a much steeper slope for vanadate-treated infected cells would be expected. However, the result in Figure 3B indicates that the virus-induced suppression of phosphorylation is not compensated by phosphatase inhibition and consequently no virus-activated phosphatase appears to be involved. In support of these data, phosphatase assays revealed that the overall activity of tyrosine phosphatases in infected cells was not elevated compared to uninfected cells. This is indicated by constant levels of free phosphates released from two different phospho-peptides that represent common tyrosine phosphatase substrates (Figure 3C). Thus, involvement of phosphatases in influenza virus-induced alteration of STAT1 phosphorylation can be greatly ruled out.

### Influenza virus infection results in the induction of SOCS-3 mRNA expression

**Figure 4:**
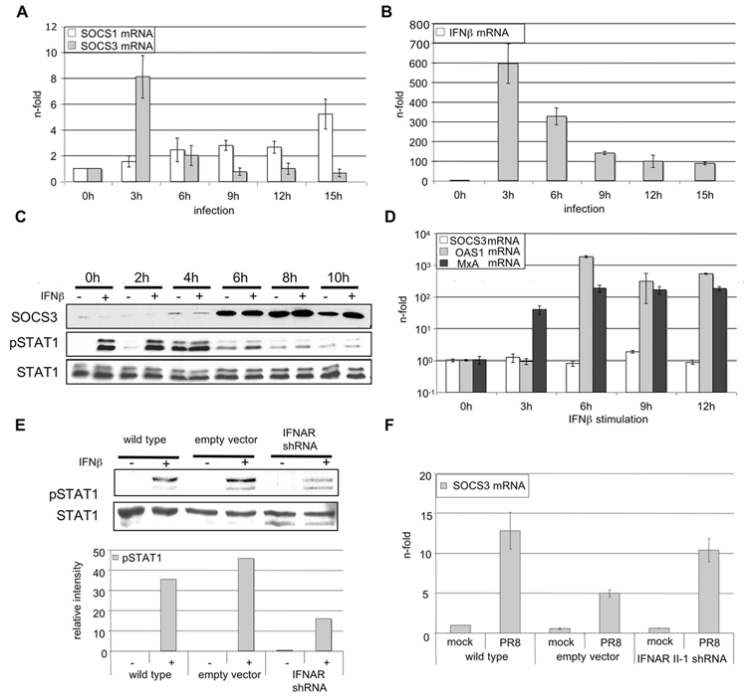
Influenza A virus results in early SOCS-3 gene induction in an IFNβ-independent manner. A549 cells were infected with PR8 (MOI = 5) (A, B, C) or stimulated with 100 U/ml human IFNβ (D) for the indicated time points. (F) A549 cells stably expressing IFNAR II-1 mRNA specific shRNA or control empty vector were infected with PR8 (MOI = 5) for 3 hours. Cells were lysed and RNA was subjected to reverse transcription. cDNA was analyzed in quantitative real time PCR to assess mRNA amounts of IFNβ (B), SOCS-1 (A), SOCS-3, (A, D, F), OAS1 (D) or MxA (D). Equivalent mRNA amounts were normalized to GAPDH mRNA levels and calculated as n-fold of the levels of untreated cells that were arbitrarily set as 1. To detect SOCS-3 protein expression (C) cells were infected for time points indicated or left uninfected. Total cell lysate was subjected to Western blot analysis using anti-SOCS-3 antibody. To allow better comparison of SOCS-3 protein expression and STAT1 phosphorylation phospho-STAT1 and STAT1 Western blots from figure 1A are shown again here. (E) To functionally test effective knock down of the IFNAR, A459 wild type, A549 vector control cells or A549 cells stably expressing IFNAR II-1specific shRNA were stimulated with human IFNβ (100 U/ml) for 15 min. Subsequently cells were lysed and levels of phospho-STAT1 were determined by Western blotting using specific antibodies. In addition, the relative pSTAT1 band intensities were quantified.

Phosphorylation of STATs in the IFNβ signaling cascade may not only be counter-regulated by phosphatases but also by other cellular factors, such as proteins of the suppressors of cytokine signaling (SOCS) family. Action of these proteins is mainly controlled on the level of transcriptional activation. SOCS proteins are described to have high affinity for JAK and STAT proteins and to inhibit the transmission of IFNα and IFNβ induced signaling [44],[45]. To examine whether expression of SOCS genes is induced in influenza virus infected cells, A549 cells were infected with PR8 for different time points. Subsequently total RNA was analyzed for the amount of SOCS-1 and SOCS-3 mRNA by means of quantitative real time-PCR (qRT-PCR). The mRNA levels of SOCS-1 and SOCS-3 differed notably in the time course (Figure 4A). While SOCS-3 mRNA is strongly and transiently elevated in the early phases of infection, SOCS-1 gene transcription is only significantly induced 15 h p.i.. Elevated SOCS-3 mRNA levels were also observed in other host cell types, such as HUVEC starting 3 h p.i. (Figure S1A). Although elevation of SOCS-3 mRNA levels in infected cells was rather transient, there appears to be a robust induction on protein level (Figure 4C). First detected at 4 h p.i., SOCS-3 protein levels increased and stayed on a high level throughout the observation period. Strikingly, expression kinetics of the SOCS-3 protein perfectly matched the kinetics of virus-induced inhibition of STAT1 phosphorylation (Figure 4C), indicating that both processes are functionally linked.

### Early induction of SOCS-3 gene transcription is not indirectly mediated by IFNβ

Virus mediated SOCS-3 gene induction at early stages of infection (Figures 4A, 4C and S1A) appeared to occur concomitant with an immediate and strong induction of IFNβ (Figures 4B and S1D). This prompted us to analyze whether SOCS-3 transcription might be induced due to an auto- or paracrine action of IFNβ expressed during infection. A549 cells were stimulated with IFNβ for different time points and SOCS-3 gene induction was measured by qRT-PCR (Figure 4D). As a control we monitored expression of 2′, 5′ -oligoadenylate synthetase (OAS1) and MxA, genes that are typically induced by IFNβ. While OAS1 and MxA mRNAs were readily upregulated upon IFNβ treatment SOCS-3 mRNA was not significantly elevated (Figure 4D). Similar results were obtained from HUVEC stimulated with IFNβ (Figure S1E). To further confirm these results we knocked down the IFNAR in A549-cells by an siRNA approach. Although the knock down was efficient and leads to more than 60% inhibition of IFNβ induced STAT1 phosphorylation (Figure 4E), the induction of SOCS-3 expression was not impaired (Figure 4F). SOCS-3 levels in the knock down cells were similar compared to wild type cells and even higher than in the vector control (Figure 4F). These results are consistent with data gained from previous experiments in Vero cells (Figure 1E) and indicate that neither induction of SOCS-3 mRNA nor inhibition of STAT phosphorylation is dependent on virus-induced type I IFN expression.

### Viral 5′ triphosphate RNA is the major inducer of SOCS-3 gene transcription and causes reduced STAT1 phosphorylation

**Figure 5:**
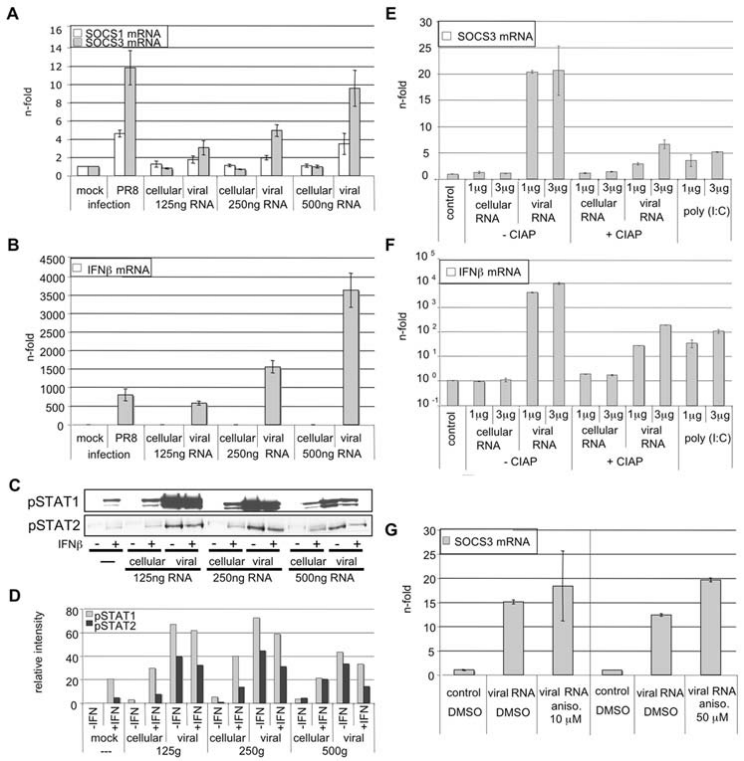
Viral 5′ triphosphate RNA efficiently induces SOCS-3 expression. Total RNA from infected or uninfected A549 cells was isolated and used for transfection of native A549 cells with L2000 according to manufacturer's instructions (A–G). Transfection of RNA from infected cells (“viral RNA”) serves as a mimic for vRNA accumulation in infected cells while total cellular RNA from uninfected cells (“cellular RNA”) was used as a control. In (E and F) different amounts of poly (I:C) or RNA from infected or uninfected cells treated with phosphatase (CIAP) as indicated were transfected using L2000. In (G) viral RNA transfected cells were additionally treated with DMSO (solvent) or the protein synthesis inhibitor anisomycin (aniso.) at the concentrations indicated. (A, B, E, F, G) Cells were lysed 3 h post transfection and total RNA was reverse transcribed. cDNA was analyzed in quantitative real time PCR to assess amounts of SOCS-1 (A), SOCS-3 (A, E, G) and IFNβ (B, F) mRNA levels. Equivalent amounts of mRNA were normalized to GAPDH mRNA levels and calculated as n-fold of untreated cells, arbitrarily set as 1. In (C) cells were treated as in (A) and (B) and monitored for phospho-STAT1 and phospho-STAT2 levels in Western blot analysis. (D) Quantification of relative phospho-STAT1 and phospho-STAT2 band intensities in (C).

Since accumulation of viral RNA in infected cells is a potent inducer of antiviral gene expression we investigated its ability to induce SOCS-3 gene transcription. As a source for viral RNA, A549 cells were infected with influenza A virus for 10 h and total RNA from these cells was isolated. RNA from uninfected A549 cells served as a negative control. Different amounts of these RNAs were used for stimulation of A549 cells for 3 h (Figures 5A, 5B and 5C). Transfection of RNA from uninfected cells did not result in an increase of SOCS-1 or SOCS-3 gene transcription (Figure 5A) or IFNβ induction as a control (Figure 5B). However, transfection of RNA from virally infected cells led to strongly elevated SOCS-3 mRNA amounts while SOCS-1 mRNA is only induced weakly (Figure 5A). This dose dependent induction of SOCS-3 by stimulation with increasing amounts of RNA from infected cells corresponds with a gradual decrease in the ability of this RNA to induce or potentiate STAT1/2 phosphorylation (Figure 5C).

In contrast to cellular RNA, influenza viral RNA carries a triphosphate group at its 5′ terminus that was previously shown to be a major pathogen pattern that triggers cellular signaling [46]. To verify that indeed the viral 5′ triphosphate RNA in the pool of RNAs from infected cells is the major trigger for induction of SOCS-3 expression, RNA from infected or uninfected cells was treated with phosphatase to remove the 5′ triphosphate termini prior to stimulation of A549 cells (Figure 5E and 5F). The dephosphorylated viral RNA was only poorly capable to induce SOCS-3 (Figure 5E) or IFNβ (Figure 5F) mRNA expression. In addition, poly(I:C) was transfected to mimic action of double-stranded (ds) RNA (Figure 5E and 5F, right bars). However, the dsRNA analog showed surprisingly little effects on SOCS-3 and IFNβ mRNA induction.

Since viral RNA is able to induce IFNβ gene transcription (Figure 5B and 5F) we again wanted to rule out that induction of SOCS-3 by viral 5′ triphosphate RNA is mediated by auto- or paracrine action of *de novo* synthesized IFNβ. In order to do so, cells were stimulated with viral RNA after treatment with the protein synthesis inhibitor anisomycin at two different concentrations (Figure 5G). SOCS-3 mRNA was still induced to the same extent in the presence of the protein synthesis inhibitor, providing the ultimate proof that *de novo* protein synthesis is not required for SOCS-3 induction.

### SOCS-3 gene transcription involves the NF-κB signaling pathway

**Figure 6:**
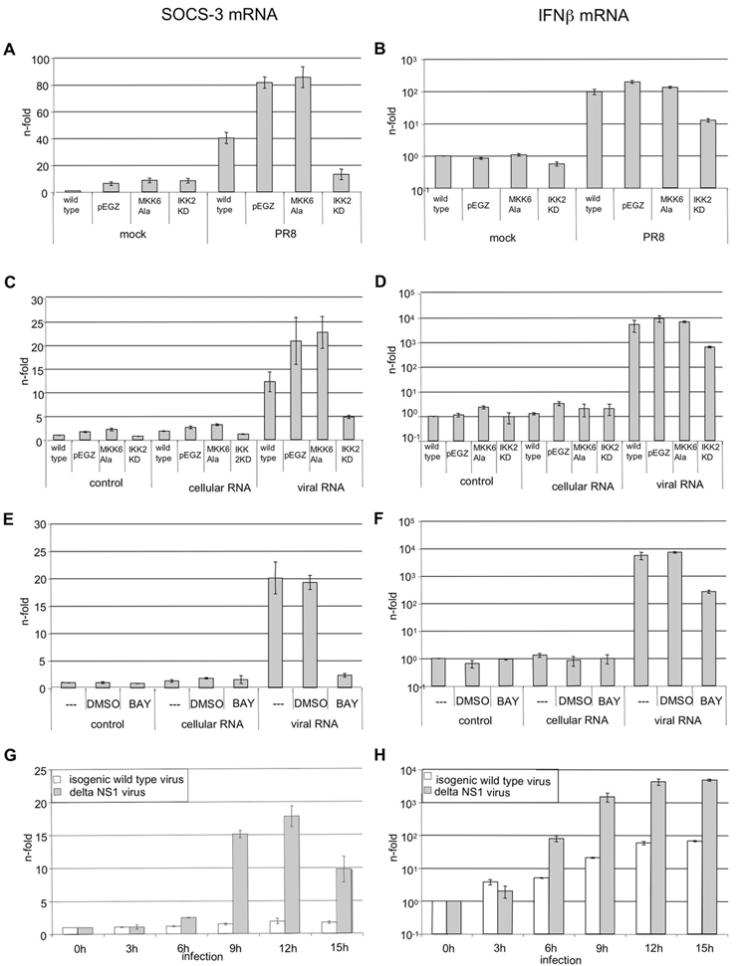
SOCS-3 mRNA transcription is induced in an NF-κB dependent manner. A549 wt cells or A549 cells stably transduced with empty vector, dominant negative MKK6Ala or IKK2KD were either infected with PR8 for 3 hours (MOI = 5) (A and B) or with the influenza A virus mutant ΔNS1 and the corresponding isogenic wild type virus (G and H) [74] or transfected for 3 hours with RNA from infected or uninfected A549 cells (C–F). (E and F) A549 cells were treated with 40 µM of the NF-κB inhibitor BAY 11-7085 30 minutes prior transfection of RNA from infected (“viral RNA”) or uninfected A549 wt cells (“cellular RNA”). In all experiments shown total RNA from target cells was isolated and reverse transcribed. cDNA was subjected to quantitative real time PCR. mRNA levels of SOCS3 (A, C, E, G) or IFNβ (B, D, F, H) were assed by specific primers.

So far, our data suggest that influenza virus-induced transcriptional upregulation of the SOCS-3 gene is not mediated by the autoregulatory action of type I IFNs (Figure 4D and 4F) but is directly induced through accumulation of viral RNA during infection. This raises the question, which RNA-induced signaling pathways are responsible for SOCS-3 expression. The MKK/p38 mitogen activated protein kinase (MAPK) pathway [47]–[49] as well as the IκB kinase (IKK)/nuclear factor of κB (NF-κB) cascade [50]–[52] are both known to be activated by RNA or influenza virus infection and to be involved in the control of SOCS-3 expression. To assess whether the MKK6/p38- or the IKK/NF-κB-module is required for SOCS-3 gene induction, we generated A549 cell lines expressing dominant negative forms of either MKK6 (MKK6Ala) or IKK2 (IKK2KD) (Figure 6A to 6D). These mutants have been previously shown to efficiently block p38 or NF-κB signaling, respectively [52]–[54]. To monitor SOCS-3 gene induction, wild type, vector or mutant expressing cell lines were infected with PR8 (Figure 6A) or stimulated with RNA from virally infected or uninfected A549 cells (Figure 6C). Induction of IFNβ mRNA was monitored as a control (Figure 6B and 6D). While MKK6Ala expression did not result in significant reduction of SOCS-3 in either infected (Figure 6A) or RNA-stimulated cells (Figure 6C), transcription is markedly reduced in IKK2KD expressing cell lines. To obtain independent evidence for NF-κB dependence of SOCS-3 gene transcription, A549 wild type cells were incubated with the NF-κB specific inhibitor BAY 11-7085 prior to stimulation with RNA from virally infected or uninfected A549 cells (Figure 6E). Again, IFNβ mRNA levels were assessed for control purposes (Figure 6F). Both, SOCS-3 and IFNβ mRNA levels were strongly reduced in BAY 11-7085 treated cells. This indicates that virus-induced SOCS-3 expression strongly depends on IKK2 and NF-κB activation, while the MKK6/p38 appears not to play a prominent role.

To further verify that influenza virus induces SOCS-3 via an RNA sensory pathway and in an NF-κB dependent manner we infected cells with the influenza A virus mutant deficient for NS1 (ΔNS1) (Figure 6G and 6H). The NS1 protein is known to block RNA dependent signaling and NFκB activation [55]. Accordingly, infection of cells with the mutant virus resulted in a more pronounced and sustained, albeit delayed induction of SOCS-3 (Figure 6G) if compared to infection with the isogenic wild type, that is a very poor inducer of SOCS-3 but still reasonably well induces IFNβ. Noteworthy, this isogenic wild type strain differs from the PR8 wild type virus used in the other experiments shown here (see Materials and Methods for details).

To analyze whether NF-κB activation is sufficient for SOCS-3 gene induction we stimulated cells with IL-1β (Figure S2A) or TNFα (Figure S2B) that are both strong activators of the transcription factor. While mRNA levels of IL-6, a strictly NF-κB dependent cytokine, are strongly elevated, SOCS-3 gene transcription is not significantly induced. Under the assumption that these cytokines do not additionally induce counteracting processes one can conclude that NF-κB is required, yet not sufficient for the induction of SOCS-3. Thus viral induction of SOCS-3 may require additional factors that are only active in virus-infected cells. Furthermore, these results rule out a potential role of virus-induced IL-1b or TNFα in the induction of SOCS-3. This is supported by the observation that neither expression of IL-1b (Figure S2C) nor TNFα (not shown) is significantly induced upon virus infection.

### SOCS-3 knock out results in enhanced constitutive STAT1 phosphorylation, and enhanced virus-induced expression of ISGs

**Figure 7:**
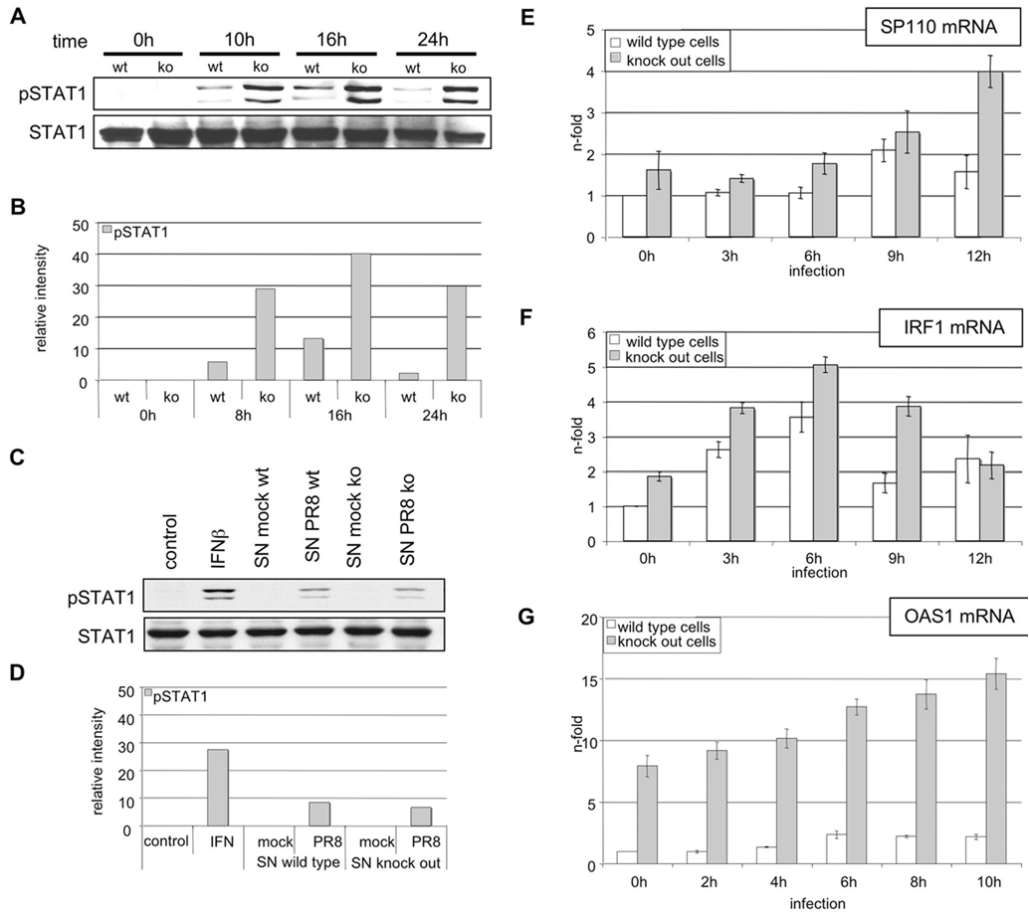
Enhanced STAT1 phosphorylation in infected SOCS-3 deficient MEF correlates with elevated induction of IFNβ-stimulated genes. Wild type MEF and SOCS-3 knock out MEF were infected with PR8 (MOI = 5) for the indicated times. Subsequently, cell lysates were analyzed for STAT1 phosphorylation (A). For control of productive virus replication, cell lysates were analyzed for viral protein PB1 expression. In (E, F, G) wild type and knock out cells were lysed at indicated time-points of infection. Subsequently RNA was subjected to reverse transcription. cDNA was analyzed in quantitative real time PCR to assess mRNA amounts of three prototype type I IFN-stimulated genes, SP110 (E), interferon regulatory factor-1 (IRF-1) (F) and OAS1 (G). Equivalent mRNA amounts were normalized to GAPDH mRNA levels and calculated as n-fold of the levels of untreated cells that were arbitrarily set as 1. In (C) wild type MEF and knock out MEF were infected with PR8 (MOI = 5) or left uninfected. Supernatants were taken 6 p.i. and used for stimulation of wild type MEF for 15 minutes. As control wild type MEF were stimulated with 500 U/ml mouse IFNβ for 15 minutes. Cells were harvested and analyzed for the amount of STAT1 and phospho-STAT1 in Western blot analysis by specific antibodies. In (B) and (D) the relative band intensities of phospho-STAT1 of the blots in (A) and (C) were quantified as described.

To further assess a functional role of SOCS-3 in virus-induced suppression of STAT1 phosphorylation we analyzed mouse cells with a targeted deletion of the SOCS-3 gene [56]. Wild type and SOCS-3 deficient mouse embryonic fibroblasts (MEF) were infected for different time points with PR8. The time of infection was prolonged in comparison to the infection of A549 cells because the human PR8 replicates less efficiently in mouse than in human cells. Following infection lysates of these cells were assessed for STAT1 phosphorylation (Figure 7A). Both cell types showed no phosphorylation of STAT1 in the uninfected state. In contrast, infection of SOCS-3 knock out cells resulted in strongly elevated phosphorylation of STAT1 in a sustained fashion. To rule out that this STAT1 phosphorylation is due to altered secretion of IFNβ or other STAT1-activating cytokines in SOCS-3 deficient cells, we performed conditioned medium experiments (Figure 7C). MEF wild type and MEF SOCS-3 deficient cells were infected for 6 h and supernatants were subsequently harvested. Stimulation of MEF wild type cells with these different supernatants for 15 min. revealed no differences in STAT1 phosphorylation, indicating that both infected cell types secrete similar amounts of IFNβ and other STAT1 activating cytokines. This is a strong indication that the observed differences in virus-induced STAT phosphorylation are directly due to the presence or absence of SOCS-3 in wild type and knock out MEF, respectively. To answer the question whether enhanced STAT phosphorylation in SOCS-3 deficient cells would also lead to enhanced expression of ISGs, total RNA was isolated at different time points p.i. from infected wild type and knock out cells and monitored for induction of SP110, IRF-1 and OAS1 (Figure 7E, 7F and 7G). These genes are described as type I IFN-induced genes [18]. Indeed mRNA levels of all three representative ISGs were elevated in SOCS-3 knock out versus wild type cells at almost every time point during the course of infection. This indicates that enhanced STAT1 phosphorylation and activation in SOCS-3 deficient cells results in elevated expression of ISGs.

### Efficiency of viral propagation is affected by SOCS-3 expression levels

**Figure 8:**
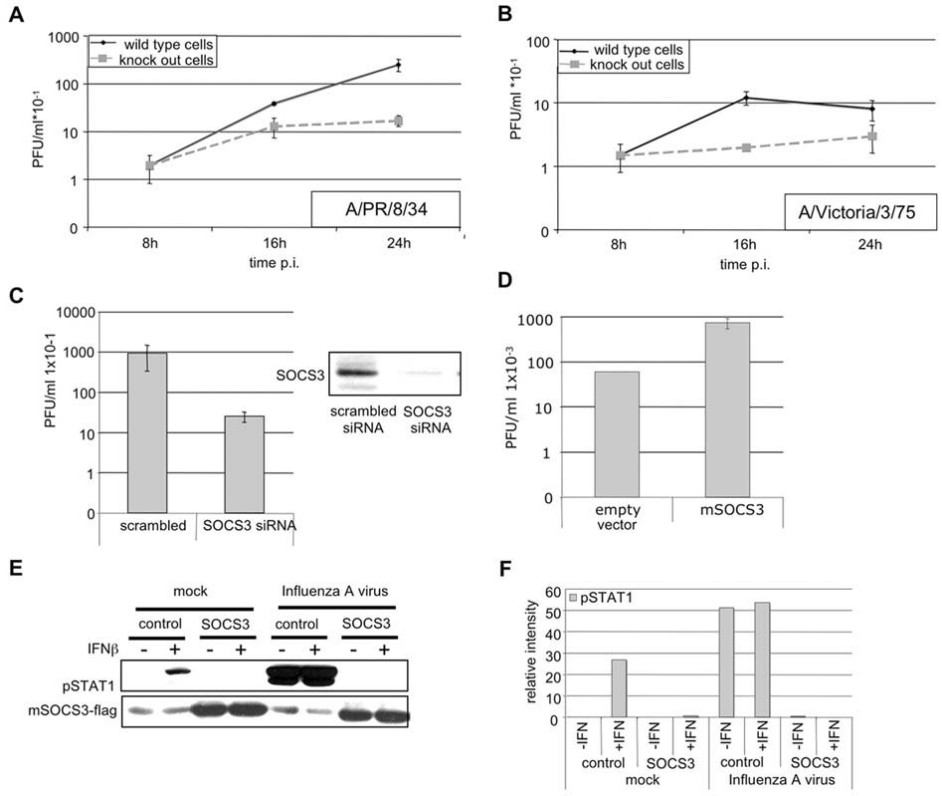
Efficiency of influenza A virus replication is dependent on SOCS3 expression levels. Wild type MEF and SOCS-3 knock-out MEF were infected with PR8 (MOI = 0.01) (A) or A/Victoria/3/75 (MOI = 0.001) (B) for the indicated times. In (C) A549 wt cells were transfected for 48 h with 150 nM human SOCS3 siRNA using Hiperfect according to manufacturers protocol and infected with PR8 (MOI = 0.001) for 9 h. In (D) the highly susceptible cell line HEK293 was transfected with either pSUPER empty vector or pSUPER-mSOCS-3 for 48 h. Subsequently cells were infected with PR8 (MOI = 0.001) for 9 h. For (A), (B), (C) and (D) progeny virus titers were determined from the supernatants of infected cells by means of plaque assay. To determine the effect of over expressed SOCS-3 on STAT1 phosphorylation (E) A549 cells were treated as in (D) and infected with PR8 (MOI = 5) and/or stimulated with human IFNβ (100 U/ml). Cells were lysed and cell extracts were analyzed for levels of phosphorylated STAT1 and over expressed mSOCS-3 using anti phospho-STAT1 and anti flag-antibody in Western blots. Effective of SOCS-3 knock down was determined by Western blot (C, right). Cells were treated as in (C, left) and total cells lysates were analyzed for endogenous SOCS-3 protein levels using anti-SOCS-3 antibody in Western blot. (F) Quantification of relative pSTAT1 band intensities in (E).

The remaining question was, whether the elevated IFN-induced gene response in knock out cells might also affect propagation of influenza A viruses. Thus, both wild type and knock out cells were infected with PR8 (Figure 8A) or the strain A/Victoria/3/75 (H3N2) (Figure 8B). Virus titers were assessed at different time points post infection. Progeny virus titers from SOCS-3 knock out cells were significantly reduced compared to titers from infected wild type cells. To independently confirm these results and to verify that the observed effects are really due to the lack of SOCS-3, we used an siRNA approach to specifically knock down SOCS-3 mRNA in A549 cells. Cells were transfected with 150 nM siRNA for 48 h and SOCS-3 protein levels were compared to control transfected samples (Figure 8C, right). Subsequently, cells were infected and progeny virus titers were determined by plaque assay (Figure 8C, left). Similar to the results gained from infected knock out cells, knock down of SOCS-3 resulted in decreased virus titers.

On the contrary, over-expression of SOCS-3 resulted in elevated virus titers (Figure 8D) concomitant with an inhibition of IFNβ- or virus-induced STAT1 phosphorylation (Figure 8E).

Taken together the data indicate that in the absence of SOCS-3, infection leads to a stronger activation of STAT1, resulting in enhanced expression of ISGs and reduced virus titers. Vice versa, over-expression of SOCS-3 leads to an inhibition of STAT1 activation and elevated virus titers, probably due to inhibited expression of ISGs.

This highlights the important role of virus induced SOCS-3 to limit the type I IFN-induced antiviral response program.

## Discussion

The type I interferon (IFN) system is one of the most powerful innate defenses of vertebrate cells, which limits replication and spread of viral pathogens including avian and human influenza viruses. Influenza virus propagation is sensitive to IFN activities and therefore, like other viral pathogens, these viruses do not only induce type I IFN but also antagonize the production and effects of these cytokines at the same time [55]. For influenza A and B viruses, this is accomplished through their non-structural NS1 proteins that are structurally related polypeptides of 26 kDa (A/NS1) and 32 kDa (B/NS1), which are abundantly expressed in infected cells [55]. NS1 proteins predominantly act on the level of IFN gene induction in infected cells by obstructing RIG-I-dependent signaling through interaction with cellular factor(s) and/or sequestration of RNAs generated during virus replication [1],[2],[57]. Some NS1 proteins were also described to inhibit the maturation of cellular pre-mRNAs raising the possibility that this activity additionally reduces production of IFNα/β in infected cells [58],[59]. While NS1 also interferes with the activity of some ISGs, such as the dsRNA dependent kinase PKR [5],[60], so far no type I IFN antagonistic mechanism was described for influenza viruses that act on the level of IFN signaling rather than gene induction. Here we present data, showing that RNA-induced expression of SOCS-3 in early phases of infection leads to a functional inhibition of IFN-induced STAT activation and gene expression. This is a novel mechanism by which influenza virus suppresses the antiviral response of the host and paves the path to efficient virus replication.

While it was reported in the literature that expression of SOCS proteins can be induced upon stimulation with IFN [61] we could not detect any significant gene induction by IFNβ in A549 cells. Instead we observed a significant up-regulation of SOCS-3 by viral 5′ triphosphate RNA, indicating that gene induction occurs via accumulation of vRNA during infection. This appears to occur through the RNA-mediated activation of the IKK/NF-κB pathway, most likely activated through engagement of the RNA sensor RIG-I. At a first sight, this might appear controversial since NF-κB activation is among the RIG-I-induced signaling responses and NS1 was reported to inhibit this signaling pathway. However, despite the action of NS1 it is well known that NF-κB is still significantly activated upon influenza virus infection and many NF-κB and IFNβ dependent genes are still expressed. We hypothesized previously that the incomplete inhibition conferred by NS1 is an indication that the virus exploits the remaining signaling activities for efficient replication [52],[62],[63]. The findings described here, namely NF-κB dependent induction of SOCS-3 and limitation of type I IFN signaling responses, provide yet another example how influenza viruses take advantage of NF-κB activity.

While the data show that NF-κB is required for viral SOCS-3 induction, the factor appears not to be sufficient, since prototype inducers of NF-κB, such as IL-1β or TNF-α would not induce SOCS-3. Thus there seems to be the need of additional virus or RNA-induced transcription factors. The most likely candidate would be the constitutively expressed interferon regulatory factor 3 (IRF-3), that is known to be simultaneously activated with NF-κB upon virus infection directly via the RIG-I RNA sensing pathway without the need of type I IFN. Furthermore IRF-3 is a factor suppressed by the NS1 protein.

Recently it was reported that IFN-induced gene expression responses are potentiated in cells, which lack the NF-κB factors p50 or p65 [64]. Although these authors described an inhibitory binding of NF-κB transcription factors to some IFN-induced gene, this mechanism might be cell type dependent since we could not observe similar effects in the cell types used here (data not shown). Thus, the underlying molecular mechanisms appear to be not fully clear. It is striking that the effects described for p50 and p65 knock out cells in these studies fully correlate with our observations in SOCS-3 deficient cells. While in the latter case cells lack the IFNβ signaling inhibitor SOCS-3, the p50 and p65 knock out cells are deficient for the factors required for SOCS-3 induction. Thus, given the NF-κB dependent induction of SOCS-3 described in the present manuscript, we provide an additional molecular mechanism that may explain the phenomenon described by Wei et al. [64].

First indications for beneficial effects of SOCS-3 gene expression on viral replication came from studies using the HCV core protein as a replacement for the influenza A viral NS1 in the context of infections with a NS1 deficient influenza virus [33]. One of the hallmark responses of HCV core expression is a rapid induction of SOCS-3 expression. Given the role of SOCS-3 described here, it was not surprising that HCV core could partially rescue growth of the NS1 deficient virus [33].

While this manuscript was in preparation it was demonstrated by Pothlichet et al. that influenza A virus-induced SOCS-1 and SOCS-3 upregulation requires a TLR-3-independent, RIG-I/MAVS-dependent pathway [65]. Moreover, over-expression of SOCS-1 and SOCS-3 in infected cells revealed that both molecules inhibit antiviral responses. These studies are perfectly complemented by our findings. Here we confirm involvement of RIG-I/MAVS by showing that 5′ triphosphate RNA, the ligand for RIG-I, is a major inducer of SOCS-3. Furthermore, the finding that dsRNA is only a weak inducer of SOCS-3 is also consistent with the independence from the dsRNA sensor TLR-3. The only discrepancy of this work and the study of Pothlichet et al. is that they show a dependence on the type I IFN receptor. This may be due to the different virus-strains and cell types used. It is well known that the capability of type I IFNs to induce SOCS proteins is strongly cell type specific [31]. While in some cell types SOCS-3 expression appears to be type I IFN dependent (e.g. fetal liver cells) [31] it is clearly independent of IFN in other cell types [66]. Recently it was shown that SOCS-3 is not significantly induced by IFNα in A549 cells [18], the major cell type used in our study. Evidence that cell type specificities may be the cause of discrepancy is additionally provided by the fact that Pothlichet et al. show identical induction kinetics of SOCS-1 and SOCS-3. In contrast the kinetics of the two proteins differ clearly in the cells we used, with SOCS-3 being induced much earlier than SOCS-1 on mRNA and protein level.

Finally, it should be stated that regardless whether SOCS-3 is additionally induced by type I IFNs at a later stage of infection, it is important that it can be induced earlier and in parallel to IFNβ directly by vRNA accumulation. This is supported by the finding that IFNβ and SOCS-3 induction occurs in parallel kinetics (Figure 4A and 4B) while IFN-induced genes such as OAS1 and MxA are only up-regulated later in a delayed and more sustained fashion (Figure 4D). This makes a qualitative difference since the blocking effect of SOCS-3 on IFNβ signaling already kicks-in during the first wave of IFNβ action.

Taken together we describe here for the first time that at least some influenza A virus strains are able to suppress type I IFN signaling by a mechanism involving NF-κB dependent activation of SOCS-3 expression, which negatively affects STAT phosphorylation. This adds a new aspect to our knowledge of the strategies used by influenza A virus to antagonize type I IFN responses.

## Materials and Methods

### Cell culture, viruses and infection conditions

Human influenza A/Puerto-Rico/8/34 (H1N1) (PR8) (Giessen variant) and A/Victora/3/75 (H3N2) (Victoria) were originally taken from the strain collection of the Institute of Virology, Giessen, Germany. The human NS1 deficient influenza virus mutant ΔNS1 and its isogenic wild type variant were propagated and used as described earlier [7],[67]. It should be noted that this isogenic wild type strain as described by Garcia-Sastre et al. [68] is different from the PR8 (Giessen variant) used in the other experiments and in many previous studies [52],[67]. While both variants are of the PR8 Mount Sinai type they exhibit different replication properties. The human alveolar epithelial cell line A549 and the corresponding A549 IFNAR II-1 shRNA and A549-nt-pLKO.1-2 cells (generated at the RKI, Berlin, Germany), as well as the human embryonic kidney (HEK) cell line HEK293, the green monkey epithelial cell line Vero and mouse embryonic fibroblasts (MEF) were grown in Dulbecco's minimal essential medium (D-MEM). Madin-Darby canine kidney (MDCK) cells were grown in minimal essential medium (MEM). All growth media contained 10% heat-inactivated fetal bovine serum and antibiotics. Human umbilical vein embryonic cells (HUVEC) were grown in endothelial growth medium (EGM, Lonza). For infection, cells were washed and infected with the multiplicity of infection (MOI) as indicated in the figure legends. For infection PBS/BA [PBS containing 0.2% bovine serum albumin (BSA), 1 mM MgCl2, 0.9 mM CaCl2, 100 U/ml penicillin, 0.1 mg/ml streptomycin] and virus were incubated for 30 minutes at 37°C. The supernatant was aspirated and cells were incubated with specific medium containing 0.2% BSA and antibiotics. To score for production of viral plaques the overlay was stained for 1 h using 1 ml neutral red in PBS per well [69].

### Stimulation of cells with cytokines and /or treatment with sodium vanadate

To trigger JAK/STAT signaling cells were stimulated using human IFNα/β or γ as well as mouse IFNβ. For stimulation of A549 cells or HUVECs 100 U/ml human IFNα or human IFNβ was used. For stimulation of the green monkey epithelial cell line Vero or HEK 293 cells 500 U/ml human IFNβ was applied. IFNγ was always used in the concentration of 500 U/ml. Mouse embryonic fibroblasts (MEF) were incubated with 100 U/ml mouse IFNβ. The different IFN were diluted in infection medium. For stimulation after infection, viral supernatants were aspirated and diluted cytokine was incubated for 15 minutes at 37°C. To investigate the potential of other cytokines to induced SOCS-3 gene expression A549 cells were stimulated with 100 U/ml IL1β or 20 ng/ml TNFα at 37°C for times indicated. After stimulation cells were lysed and subjected to immune blotting.

To block the activity of phosphatases after infection with influenza virus, sodium vanadate was used. Dilutions were prepared using infection medium. Sodium vanadate was added to the virus-containing infection medium at the time points indicated. After 10 minutes of incubation IFNβ, diluted in infection medium, was added to the medium containing virus and sodium vanadate. The cells were stimulated with IFNβ for 15 minutes. Incubation with sodium vanadate started 25 min before cells were lysed and subjected to Western blotting as described.

For conditioned medium experiments wild type and SOCS-3 knock out MEF were infected with PR8 (MOI = 5) for 10 h or left uninfected. Supernatants were used for stimulation of MEF wild type for 15 minutes. Cell lysates were subjected to Western blot analysis.

### Stimulation of cells with phosphorylated or dephosphorylated viral or cellular RNA

To investigate the induction of SOCS-3 expression by viral RNA, RNA isolated from infected or uninfected cells (control) was used. A549 cells were infected with PR8 (MOI = 5) or left mock infected. 10 h post infection RNA was isolated using the RNeasy mini Kit from Qiagen according to manufacturer's instructions. To dephosphorylate viral 5′ triphosphate RNA, calf intestine alkaline phosphatase (CIAP) (Fermentas) was used. Briefly, RNA was isolated using Trizol according to manufacturer's instructions. For dephosphorylation the reaction mix was set up in a 50 µl volume with 50 µg RNA, 25 U CIAP and 80 U RiboLock RNase inhibitor (Fermentas) and was incubated for 3 h at 42°C. Thereafter the RNA was isolated using the RNeasy mini Kit from Qiagen. RNAs used as control were mock-treated replacing CIAP by glycerol.

For stimulation, the different RNA species and analogues were transfected using Lipofectamine 2000 (L2000) according to manufacturer's instruction (Invitrogen). In brief, L2000 was incubated with OPTI-MEM for 5 minutes at room temperature; different amounts of RNA were added and incubated for additional 15 minutes. For stimulation of cells with cellular or viral RNA 400 µl RNA-L2000 mix were added to 2 ml serum-free medium. Cells were stimulated for 3 hours and subjected to either Western blot analysis or quantitative real time PCR.

### siRNA mediated knock down of human SOCS-3

For silencing SOCS-3 mRNA, A549 cells were transfected with 150 nM human SOCS-3 siRNA 48 h before infection using Hiperfect (Qiagen) according to manufacturer's instructions. In brief, 150 nM siRNA was added to a mixture of D-MEM without FCS/antibiotics and Hiperfect and incubated for 10 min at room temperature. For transfection 400 µl of this mixture were added to the cells. Subsequently cells were subjected to plaque assay analysis or Western blot analysis. Control siRNA was purchased from Qiagen. The sequences for the human SOCS-3 siRNA in use are: human SOCS-3 siRNA sense 5′- CCA AGA ACC UGC GCA UCC AdTdT-3′, human SOCS-3 siRNA anti-sense 5′ - UGG AUG CGC AGG UUC UUG GdTdT-3′ ) (see Table 1 for accession number of the human SOCS-3 gene).

**Table 1:** Accession numbers of human, murine and viral genes used or analyzed in this study.

	Accession numbers
**Human**	
IFNβ	NM_002176.2
SOCS-3	NM_003955.3
GAPDH	NM_002046.3
OAS1 isoform 3	NM_001032409.1
OAS1 isoform 2	NM_002534.2
OAS1 isoform 1	NM_016816.2
**Murine**	
GAPDH	NM_008084.2
OAS1b	NM_001083925.1
IRF-1	NM_008390.1
SP110	NM_175397.4
**Viral**	
NP	CY009447
NS	CY009448
M	CY009445
PA	CY009449
PB1	CY009450
PB2	CY009451

### Tyrosine phosphatase assay

To determine whether tyrosine phosphatases become activated upon infection with influenza virus a phosphatase assay using the Tyrosine Phosphatase Assay System (Promega) was performed. A459 cells were infected for 10 h (MOI = 5) or left uninfected. Cells were harvested in assay buffer (100 mM tris-HCl pH 5.2, 100 mM CaCl_2_, 100 mM MgCl_2_, 0.02% β-mercapto ethanol), cracked by a single freeze/thaw step at −80°C and disrupted by ultrasonic pulsing. Lysates were precleared from cell debris and residual free phosphates according to the manufacturer's instruction. Tyrosine phosphatase activity was measured by enzymatic release of free phosphate of two given pseudosubstrates (phosphorylated peptides representing target sequences for the most common tyrosine phosphatases). Quantification was performed in comparison to a given standard according to the manufacturer's instruction.

### Western blots

For Western blot analysis cells were lysed with RIPA [25 mM Tris/HCl, pH 8.0, 137 mM NaCl, 10% Glycerol, 0.1% SDS, 0.5% NaDOC, 1% IgePal, 2 mM EDTA, pH 8.0, pyrophosphate 5 µg ml^−1^ aprotinin; 5 µg ml^−1^ leupeptin; 1 mM sodium vanadate and 5 mM benzamidine] on ice for a minimum of 30 minutes. Supernatants were cleared by centrifugation in a standard tabletop centrifuge (Eppendorf) at maximum speed. Protein concentration was determined by Bradford assay.

The phosphorylated and unphosphorylated forms of STAT1 were detected using anti-STAT1 (Y701) antibody and anti-STAT1 (BD Bioscience). An antibody directed against Y690 of STAT2 was used for detection of the phosphorylated form of STAT2 (Upstate). Antibodies to detect influenza viral proteins were purchased from Serotec (NP, M1), Santa Cruz (PB1, PB2). The anti-PA antibody was kindly provided by J. Ortin (Madrid/Spain). A monoclonal antibody directed against the viral NS1 was generated at the IMV, Muenster, Germany [70]. A monoclonal anti-Myc-tag antibody to detect Myc-M1 was kindly provided by Viktor Wixler. IMV, Muenster, Germany. All secondary antibodies were purchased from Amersham and diluted 1∶2500 in TBS-T. Secondary antibodies were incubated for a minimum of 60 minutes at room temperature.

### Reverse Transcription and quantitative real time PCR

To synthesize cDNA from cells, RNA was isolated using Qiagen RNeasy mini kit according to manufacturer's instruction. In brief, cells were lysed in the presence of β-mercaptoethanol and lysates were loaded to a column, washed and eluted in RNase- free water. For reverse transcription 3 µg total RNA, 0.5 µg oligo dT primer in a total volume of 12 µl were heated for 10 minutes at 70°C. Enzyme mix was prepared (5× Enzyme Buffer (Fermentas), water and 500 µM dNTPs) and pre-warmed at 42°C for 2 minutes before adding 535 U/100 µl RevertAid H^−^ M-MuLV (Fermentas).

Reverse transcription was performed at 42°C for 1 hour. The enzyme was inactivated at 70°C for 10 minutes. Samples were stored at −20°C or directly used in quantitative real-time PCR.

For analysis of gene expression relative quantification of the DNA amount was applied. In order to do that gene expression of the housekeeping gene GAPDH was determined. To ascertain changes in expression of the gene of interest the differences between expression of GAPDH and the gene of interest was calculated using the 2^−ΔΔCT^ method [71]. For quantitative real time Brilliant QPCR SYBR Green Mastermix (Stratagene) was used according to manufacturer's instructions. The fragment of interest was amplified in 40 cycles. The following primers were used (see Table 1 for identity of the parental genes): human primer pairs: GAPDH_fwd 5′-GCA AAT TTC CAT GG CAC CGT3′, GAPDH_rev 5′ - GCC CCA CTT GAT TTT GGA GG-3′, IFNβ-fwd 5′ - GGC CAT GAC CAA CAA GTG TCT CCT CC-3′, IFNβ_rev 5′ - GCG CTC AGT TTC GGA GGT AAC CTG T-3′, SOCS-1_fwd 5′ - TTG CCT GGA ACC ATG TGG -3′, SOCS1_rev 5′ - GGT CCT GGC CTCCAG ATA CAG -3′, SOCS-3_fwd 5′ - GGA GTT CCT GGA CCA GTA CG-3′, SOCS-3_rev 5′ - TTC TTG TGC TTG TGC CAT GT -3′, OAS1_fwd 5′ - GAT CTC AGA AAT ACC CCA GCC A-3, OAS-1_rev 5′ - AGC TAC CTC GGA AGC ACC TT-3′, MxA_fwd 5′ -GTT TCC GAA GTG GAC ATC GCA-3, MxA rev 5-GAA GGG CAA CTC CTG ACA GT-3, IL1β_fwd 5′ -GCG GCC AGG ATA TTT TAA CTG ACT TC-3, IL1β_rev 5′ -TCC ACA TTC AGC ACA GGA CTC TC-3, IL6_fwd 5′ -AGA GGC ACT GGC AGA AAA CAA C-3, IL6_rev 5′ -AGG CAA GTC TCC TCA TTG AAT CC-3′ and murine primer pairs: GAPDH_fwd 5′ - ACA GCC GCA TCT TCT TGT GCA GTG-3′, GAPDH_rev 5′ - GGC CTT GAC TGT GCC GTT GAA TTT-3, SOCS-3_fwd 5′ - GGG TGG CAA AGA AAA GGA G-3′, SOCS-3_rev 5′ - GTT GAG CGT CAA GAC CCA GT-3, IRF-1_fwd 5′ - ATG CCA ATC ACT CGA ATG CG-3′, IRF-1_rev 5′ - TTG TAT CGG CCT GTG TGA ATG-3, SP110_fwd 5′ - TAG GGA AGC ATC CAA AAC GAA TG-3′, SP110_rev 5′ - CCT GGG GCT CTT GTT CAT CAC-3′, OAS1_fwd 5′ - GTC AAT GTC GTG TGT GAT TT-3′, OAS1_rev 5′ -CTC CCC GTC GGT TTA ACT GA-3′.

### Retroviral vectors and retroviral transduction

The pCFG5-EGZ retroviral vector used for transfection [72] as well as the constructs to express dominant negative MKK6 (MKK6Ala) or IKK2 (IKK2KD) have been described earlier [52],[73]. The Phoenix amphotropic retroviral producer cells (a gift from G. Nolan, Stanford, CA) [74] were cultured in Dulbecco's modified Eagle's medium containing 10% fetal bovine serum, 100 units/ml penicillin and 100 mg/ml streptomycin. Generation of MKK6Ala or IKKKD expressing producer cells as well as transduction of A549 cells to stably express these transgenes was performed as previously described [52],[53].

## Supporting Information

Figure S1 Infection of HUVEC results in inhibition of STAT1 phosphorylation and IFNβ independent SOCS-3 gene transcription. HUVEC were infected with PR8 (MOI = 5) (A, B, D) or stimulated with 100 U/ml IFNβ (E) for time points indicated. To assess the mRNA levels of SOCS-3 (A, E), IFNβ (B) and MxA (E) RNA was reverse transcribed and cDNA was subjected to quantitative real time PCR. Equivalent mRNA amounts were normalized to endogenous GAPDH and calculated as n-fold of untreated cells that were arbitrarily set as 1. To assess the amount of phosphorylated STAT1 (B) A549 cells were infected with PR8 (MOI = 5) for time points indicated. Total cells lysate was subjected to Western Blot analysis using anti-phospho-STAT1, anti-STAT1 antibodies. To assess effective viral replication viral NS1 was detected using an anti-NS1 antibody. (C) Quantification of relative band intensities of (B) using AIDA software and 2D densitometry (Fuji).
(1.22 MB TIF)

Figure S2 IL1β and TNFα do not affect induction of SOCS-3 gene transcription. A549 wt cells were stimulated with 100 U/ml IL1β (A), 20 ng/ml TNFα (B) or infected with PR8 (MOI = 5) (C) for time points indicated. Cells were lysed, and RNA was subjected to reverse transcription. cDNA was analyzed in quantitative real time PCR to assess mRNA amounts of SOCS-3 and IL6 (A and B) or IL1β (C). Equivalent mRNA amounts were normalized to GAPDH mRNA levels and calculated as n-fold of the levels of untreated cells that were arbitrarily set as 1.
(4.87 MB TIF)
