# Supplementary figures and images for: Influenza A Virus Inhibits Type I IFN Signaling via NF-κB-Dependent Induction of SOCS-3 Expression
Source: PLoS Pathog. 2008 Nov 7;4(11):e1000196. doi: 10.1371/journal.ppat.1000196 (PMC2572141; doi:10.1371/journal.ppat.1000196)

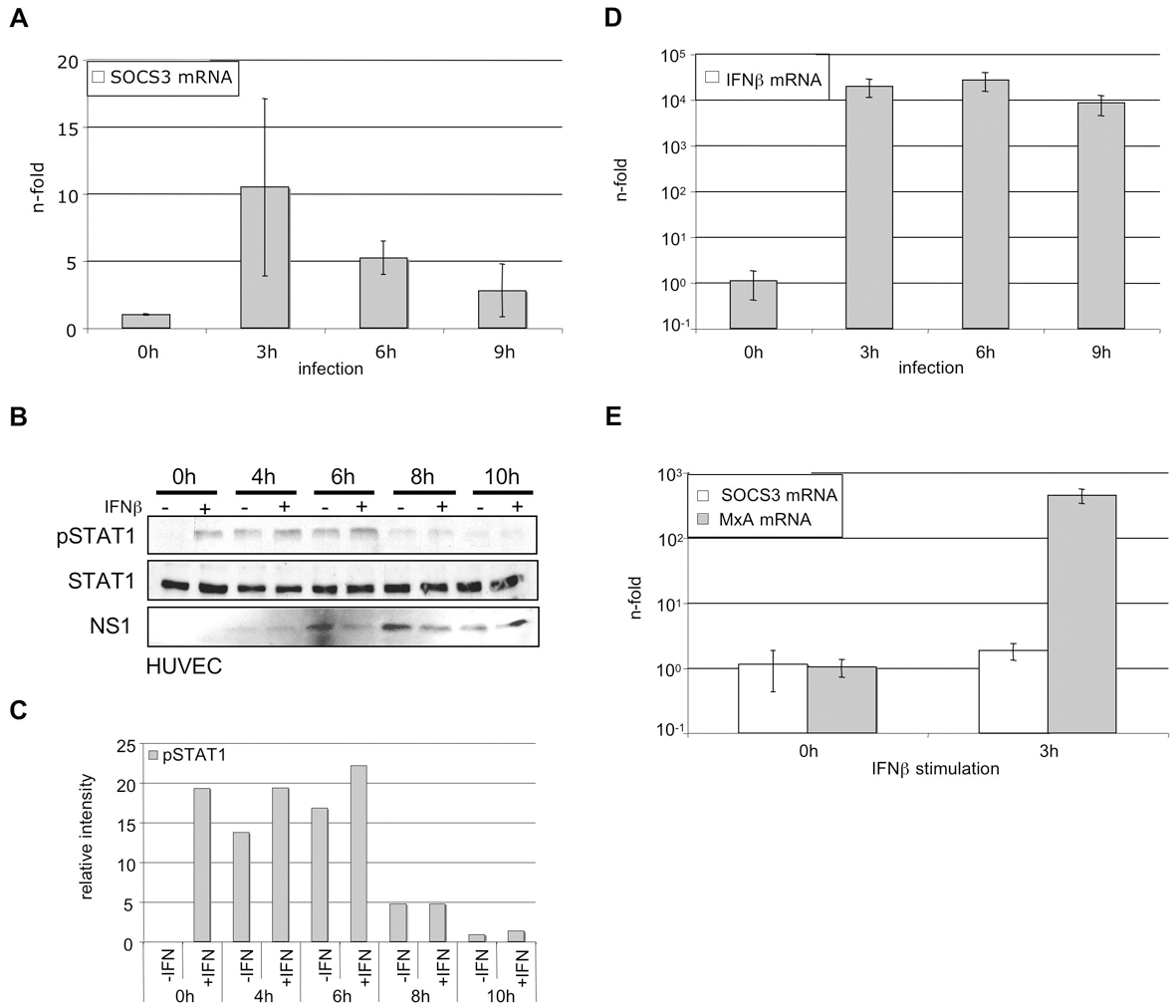

Supplement: Figure S1 — Infection of HUVEC results in inhibition of STAT1 phosphorylation and IFNβ independent SOCS-3 gene transcription. HUVEC were infected with PR8 (MOI = 5) (A, B, D) or stimulated with 100 U/ml IFNβ (E) for time points indicated. To assess the mRNA levels of SOCS-3 (A, E), IFNβ (B) and MxA (E) RNA was reverse transcribed and cDNA was subjected to quantitative real time PCR. Equivalent mRNA amounts were normalized to endogenous GAPDH and calculated as n-fold of untreated cells that were arbitrarily set as 1. To assess the amount of phosphorylated STAT1 (B) A549 cells were infected with PR8 (MOI = 5) for time points indicated. Total cells lysate was subjected to Western Blot analysis using anti-phospho-STAT1, anti-STAT1 antibodies. To assess effective viral replication viral NS1 was detected using an anti-NS1 antibody. (C) Quantification of relative band intensities of (B) using AIDA software and 2D densitometry (Fuji). (1.22 MB TIF) [file ppat.1000196.s001.tif]

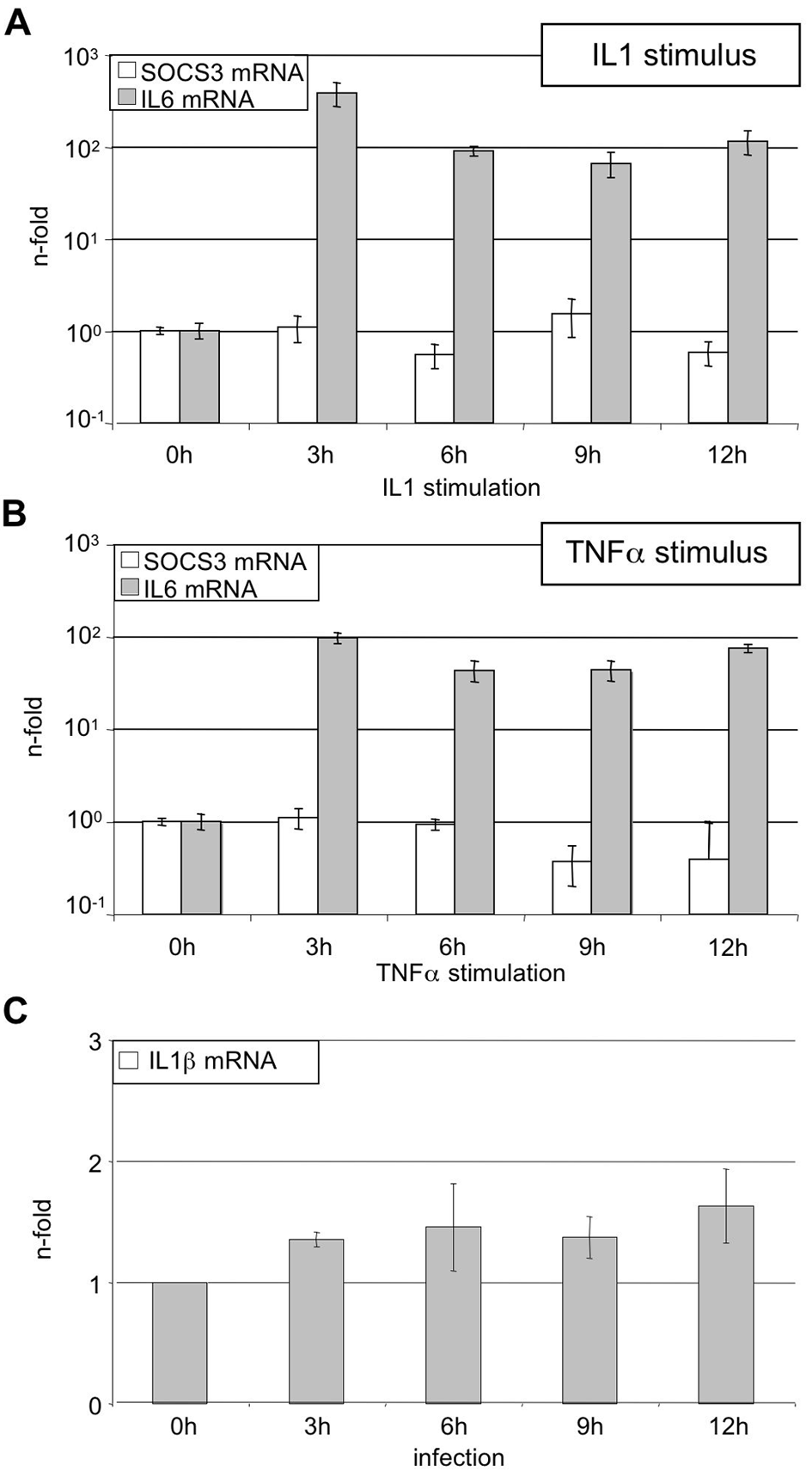

Supplement: Figure S2 — IL1β and TNFα do not affect induction of SOCS-3 gene transcription. A549 wt cells were stimulated with 100 U/ml IL1β (A), 20 ng/ml TNFα (B) or infected with PR8 (MOI = 5) (C) for time points indicated. Cells were lysed, and RNA was subjected to reverse transcription. cDNA was analyzed in quantitative real time PCR to assess mRNA amounts of SOCS-3 and IL6 (A and B) or IL1β (C). Equivalent mRNA amounts were normalized to GAPDH mRNA levels and calculated as n-fold of the levels of untreated cells that were arbitrarily set as 1. (4.87 MB TIF) [file ppat.1000196.s002.tif]
